# Styling the Self: Clothing Practices, Personality Traits, and Body Image Among Israeli Women

**DOI:** 10.3389/fpsyg.2021.719318

**Published:** 2021-09-08

**Authors:** Tali Stolovy

**Affiliations:** ^1^Academic College of Society and the Arts, Netanya, Israel; ^2^Emili Sagol Creative Arts Therapies Research Center, Faculty of Social Welfare and Health Sciences, University of Haifa, Haifa, Israel

**Keywords:** personality traits, body image, women–clothing, clothing styles, clothing practices

## Abstract

Research has shown that women tend to use clothes to present or disguise their bodies and that clothing practices can be predicted by body image. This study explored the relationships between clothing practices, personality traits, and body image among Israeli women, using the Big Five personality traits model (NEO-FFI) and a body image measure (MBSRQ) to explore clothing styles and practices among Israeli women (*N* = 792, Mean age = 42.19). It found that women with more openness to experience (OR = 1.8; IC 95%: 1.05–3.0), who seek fashion (OR = 2.05; IC 95%: 1.37–3.05) and individuality (OR = 3.96; IC 95%: 2.46–6.3) are more likely to exhibit an urban, sophisticated style of dress. These women are less motivated by comfort (OR = 0.49; IC 95%: 0.31–0.77) and camouflage (OR = 2.05; IC 95%: 1.37–3.05), that are associated with casual, minimalist style of dress. This study indicates that openness to experience may foster body-positive clothing practices. In this way, their choice of clothing can help women overcome objectification and cultural body-ideal pressures, promoting self-validation and mastery.

## Introduction

Most psychological research on clothing focuses on social and cultural perceptions of the clothing that people choose to wear. It is conducted primarily in workplaces and reflects how people perceive and judge others' professionality and reliability based on their clothes (e.g., Rehman et al., 2005; Howlett et al., 2015). The effect of clothes on the wearer is thus examined in the context of the relationship between formal or informal style of dress and the person's self-perception in the workplace (Peluchette et al., 2006).

There is a paucity of research on the idiosyncratic meanings of clothes and the emotional functions performed by daily choices of clothing—even though earlier theorists propose that clothing is the external manifestation of the self (Cooley, 1902; Flugel, 1930; Sontag and Schlater, 1982; James, 2007). Expressions such as “the second skin” and “the visible self” suggest both the physical contiguity between clothing and the body and the psychological proximity of clothing to the self.

Sontag and Lee (2004) define the psychological closeness of clothing as determined by the extent to which clothing is (1) perceived as one with the self or as a component of the self; (2) recognized as an aspect of appearance by which the self is established and validated; (3) recognized as a significant symbol of one's identity, mood, or attitude; (4) perceived as an expression of self-regard or self-worth, (5) recognized as an element of an affective response to self-evaluation; or (6) related to body cathexis.

The psychological effect of clothes on the wearers themselves was demonstrated by Adam and Galinsky's (2012) research, which found that simply donning a white lab coat increased performance on attention-related tasks and selective attention. The researchers coined the term “enclothed cognition,” which differs from embodied cognition because the link between physical experience and its symbolic meaning is indirect: it is the item of clothing that carries the symbolic meaning.

This concept of clothing's symbolic and emotional may shed light on the question: Why do people in the same social cultural environment choose certain clothing styles and not others? Kwon's (1991) research suggests that individuals' clothing choices can be a reflection of how they feel about themselves, and not only about how they want others to feel about them. Other studies have shown that individuals reinforce their mood and express their feelings through their clothing (Kallstrom, 2009). Mood can be altered because the clothes selected may be perceived as fashionable, enhancing individuality and confidence, or providing physical comfort (Kang et al., 2013). The sample of female shoppers studied by Tiggemann and Lacey (2009) primarily chose clothes for the positive functions of assurance, fashion, and comfort.

To date, psychological research on dress and clothing practices has focused almost exclusively on Western women. One rationale for this focus is that Western men have been shown to express less interest in clothing and fashion than women; millennials of all genders are likely to enjoy shopping, but women are still more involved with fashion than men (Pentecost and Andrews, 2010). However, men have come under increasing pressure to conform to the cultural ideal of a lean, well-toned, muscular body, and they, too, manage their appearance and body image through clothes (Frith and Gleeson, 2004).

The unique relationship of Western women to clothes develops on the background of their socialization into roles that are preoccupied with appearance and how others see them. Women may use clothes to display or disguise their bodies. Clothing thus affects the degree to which women are objectified and appraised (for more information on objectification theory, see Fredrickson and Roberts, 1997; Heflick and Goldenberg, 2009). Kwon and Parham (1994) found that women selected clothes more for camouflage and less for individual self-expression when they felt “fat” than when they felt thinner. Higher body mass index (BMI) and body dissatisfaction were related to the use of clothing as camouflage. Tiggemann and Andrew's (2012) findings also show interrelationships between women's attitudes toward clothing and their attitude toward their bodies.

Body image is a multidimensional construct that includes perceptual, attitudinal, and behavioral components, and dressing one's body is an intentional behavior. In other words, how individuals feel about and perceive their bodies affect how they manage their appearance through their choice of clothing (Rudd and Lennon, 2000; Jung et al., 2001). Hence, clothing practices can be predicted by body image (Tiggemann and Lacey, 2009).

Previous research on body image has also addressed personality traits. Personality is the characteristic manner in which people feel, think and behave. There is still some debate regarding the number of trait dimensions, but most scholars accept that there are at least five major dimensions of trait personality (Allen and Walter, 2016). The Five Factor Model (FFM) and Big Five Model (BFM) of personality define five higher-order domains: Neuroticism, Extraversion, Openness to experience, Agreeableness, and Conscientiousness (Goldberg, 1990; Costa and McCrae, 1992; Oliver and Srivastava, 1995). People who score higher on Neuroticism tend to be more self-conscious (Costa and McCrae, 1992), place greater importance on how they look (Davis et al., 2001), and are more likely to compare themselves to attractive others (Roberts and Good, 2010). Neuroticism was found to correlate to higher body surveillance, lower appearance control beliefs (Tylka, 2004), and higher body shame (Miner-Rubino et al., 2002). Extraversion was associated with a higher appreciation of one's own body and lower body dissatisfaction (Swami et al., 2012). Extraverts are less reserved, more assertive, and tend to be more talkative (Costa and McCrae, 1992), and these characteristics might place such individuals at a lower risk of negative body image. People who score high on extraversion also experience more positive emotions (Steel et al., 2008) and are less sensitive to social threat (Wilt and Revelle, 2008), meaning extraverted individuals might be less vulnerable to sociocultural factors that contribute to negative body image (Kvalem et al., 2006; Allen and Walter, 2016).

People who score high on conscientious tend to endorse societal conventions (Roberts et al., 2014) but are also characterized by high levels of confidence and therefore might be less receptive to exposure to idealized physical appearance (Roberts and Good, 2010).

Swami et al. (2012) reported that body appreciation was positively correlated to conscientiousness, as well as agreeableness. People who score higher on agreeableness tend to endorse traditional values (Roccas et al., 2002) and therefore assumed to assign greater importance to physical appearance, putting them at a higher risk of a negative body image (Allen and Walter, 2016). Nonetheless, Miner-Rubino et al. (2002) found that body shame was negatively correlated to agreeableness. All these studies were carried out using exclusively female samples.

People who score higher on openness tend to value intellectual and emotional autonomy, acceptance, and cultivation of diversity (Roccas et al., 2002), and therefore might be more open to different body image ideals putting them at a lower risk of negative body image (Allen and Walter, 2016).

Given that body image is correlated with personality traits and that clothing serves psychological functions (e.g., Tiggemann and Lacey, 2009) and is a reflection or expression of an individual's identity (Sontag and Lee, 2004), this study explores the relationship between clothing practices (i.e., styles of dress and clothing functions), personality traits, and body image among women. It also expands the commonly used formal and informal categories to include a wider array of clothing styles. My main research hypothesis is that clothing practices are related to personality traits and can be predicted by body image.

## Method

### Study Design

This study used a convenience sample that completed an online survey that was distributed *via* Facebook social media platform. Informed consent was provided by all participants at the beginning of the online survey. Participants were allowed to terminate the survey at any time they desired. The survey was anonymous, and confidentiality of information was assured.

The study included women all across Israel, the invitation for participants was repeated five times on social media and one time on a popular morning TV show. The questionnaire consisted of 4 parts: (1) socio-demographic data, (2) function of clothing scale, (3) MBSRQ—Body image, (4) NEO-FFI-Personality traits. It took about 15 min to complete the survey.

### Participants

The call to participate described the aim of the study and invited women to respond and complete the survey. Inclusion criteria were being over 18 years old, with no exclusion criteria. A total of 792 women completed surveys were received.

### Instruments

#### Function of Clothing Measure

Function of clothing were assessed using items developed by Kwon and Parham (1994). This scale measures the choice of clothing for its comfort, camouflage, assurance, fashion. and individuality functions. Its 20 items are assessed on a Likert scale ranging from 1 (not at all agree) to 5 (very much agree). Kang, Johnson, and Kim also used this scale in their 2013 study. It was translated to Hebrew using a translation/back translation procedure by the author and a native English speaker. In the present sample, Cronbach's alpha was 0.75. In addition to these assessments, the participants were asked to define their clothing style by choosing one of the following five options to best describe their wardrobe: (1) casual style (jeans, pants, t-shirts or cotton shirts, minimalist styling); (2) romantic style (skirts, dresses, soft fabrics, floral patterns, bohemian style, clothing that is stereotypically perceived as “feminine”); (3) dramatic style (unusual and unique outfits, bright colors and color combinations, may sometimes be tight or revealing); (4) classic style (formal clothing, conventional and representative outfits); and (5) urban or eclectic style (different combinations of all styles, mix and match, playful style of dress with combinations of low- and high-priced clothing, frequent use of accessories).

#### Body Image Measure

The Multidimensional Body-Self Relations Questionnaire (MBSRQ) is a well-validated self-report inventory for the assessment of body image that measures overall body image and satisfaction with body shape (Cash, 1994). This 34-item measure has five dimensions: Appearance Evaluation, Appearance Orientation, Overweight Preoccupation, Self-Classified Weight, and the Body Areas Satisfaction Scale (BASS). This study used all the dimensions except for the BASS subscale. Each item was scored from 1 = very dissatisfied to 5 = very satisfied. The questionnaire is characterized by a reliability of *α* = 0.78, and the Hebrew version was found reliable with Cronbach's alpha of 0.86 (Shaiovitz, 2014). In the present sample, Cronbach's alpha was 0.66.

#### Personality Traits Measure

The NEO Five-Factor Inventory (NEO-FFI; Costa and McCrae, 1992; Hebrew version by Etzion and Laski, 1998) consists of 60 items; each of the Big Five personality traits is assessed based on 12 items: neuroticism (e.g., “I often feel inferior to others”), extraversion (e.g., “I like to have a lot of people around me”), agreeableness (e.g., “I try to be courteous to everyone I meet”), openness to experience (e.g., “I have a lot of intellectual curiosity”), and conscientiousness (e.g., “I keep my belongings clean and neat”). The response format used a 4-point Likert scale, ranging from 0 (completely disagree) to 4 (completely agree). Prior research has found good cross-cultural validity of this measure in Israel (Etzion and Laski, 1998). In the present sample, Cronbach's alpha was 0.71.

#### Sociodemographic and Additional Variables

Each participant was asked to indicate her age, height and weight, country of birth, marital status, religious affiliation, educational level, health and financial status, and occupation.

### Statistical Analyses

All participants filled out the research questionnaires online through the Qualtrics website. The data were analyzed through SPSS 19.0 software. The relationship between clothing styles, clothing functions, body image and background data were analyzed using Pearson's correlations and chi-square tests. A series of one-way ANOVA analyses were used to explore the relationships of clothing style preference groups (casual, romantic, dramatic, classic, and urban) to the Big Five personality traits, body image, and functions of clothing. Finally, backwards stepwise logistic regressions were preformed to explore the effect of the different variables on each style of dress, compared with the other styles of dress.

### Ethical Considerations

Data were collected considering general ethical principles of Emily Sagol Creative art therapies research center in Haifa University. Because this concerns a study in which only adult women participate on their own free will and after informed consent, based on the ICH-GCP principles (https://www.ema.europa.eu/en/documents/scientific-guideline/ich-e-6-r2-guideline-good-clinical-practice-step-5_en.pdf) ethical approval was not sought for the present study. Informed consent was provided by all participants at the beginning of the online survey. Participants were allowed to terminate the survey at any time they desired. The survey was anonymous, and confidentiality of information was assured.

## Results

The sample comprised 792 women from urban and rural areas in Israel; the participants' ages ranged from 19 to 74 (M = 42.1, SD = 10.32). Descriptive data is presented in Table 1. As can be seen, about two-thirds of the sample were married women (*N* = 507, 64%) and mothers (*N* = 410, 51.8%). More than 80% had academic degrees (*N* = 556, 70.2%). Most of the sample considered themselves as non-religious (*N* = 572, 82.9%), and nearly half perceived themselves to be financially secure (*N* = 394, 49.7%). Chi square tests found no association between descriptive data and the main research variables.

**Table 1:** Basic information of the sample (*N* = 792).

**Variable**	**Mean, SD**
**Age**	42.19, 10.32
**Height (cm)**	164.8, 6.32
**Weight (kg)**	67.42, 12.44
**BMI**	24.8, 4.46
**Variable**	**N** ^*^ **, %**
**Marital status**	
Single	146, 18.4%
Married or living as married	507, 64%
Separated or divorced	42, 5.3%
**Children**	
Yes	410, 51.8%
No	202, 25.5%
**Education background**	
<12 years	60, 7.6%
<14 years	63, 8.0%
<16 years	493, 62.2%
**Religiousness level**	
Very religious	44, 10.7%
Religious	44, 6.4%
Not so religious	76, 11.0%
Not at all religious	496, 71.9%
**Financial status**	
Very good	108, 13.6%
Good	286, 36.1%
Moderate	200, 25.3%
Not good	16, 2.0%
Not good at all	2, 0.3%
**Health condition**	
Very good	302, 38.1%
Good	263, 33.2%
Moderate	38, 4.8%
Not good	8, 1.0%
Not good at all	1, 0.1%

* *N presented excluding missing data*.

### The Big Five Personality Traits, Functions of Clothing, and Clothing Style

The pattern of correlations among the Big Five personality traits and clothing functions is shown in Table 2. As can be seen, extroversion is moderately correlated with using clothes for assurance (*r* = 0.30, *p* < 0.001). Openness to experience is positively correlated with assurance (*r* = 0.31, *p* < 0.001) and individuality (*r* = 0.31, *p* < 0.001) and negatively correlated with camouflage (*r* = −0.24, *p* < 0.001). The remaining correlations have a weak effect below *r* = 0.20 (see Table 2).

**Table 2:** Pearson's correlations among the Big Five personality traits, body image dimensions, and clothing functions (*N* = 792).

	**Comfort**	**Camouflage**	**Assurance**	**Fashion**	**Individuality**
Extraversion	−0.01	−0.19^***^	0.30^***^	0.13^***^	0.14^***^
Neuroticism	−0.03	0.16^***^	−0.01^*^	−0.00	−0.06
Agreeableness	0.16^***^	−0.16^***^	0.15^***^	−0.09^*^	−0.05
Conscientiousness	−0.03	−0.24^***^	0.17^***^	0.09^**^	0.15^***^
Openness	0.01^*^	−0.24^***^	0.31^***^	0.07	0.31^***^
Appearance evaluation	0.01	−0.58^***^	0.34^***^	0.276^***^	0.30^***^
Appearance orientation	−0.15^***^	−0.14^**^	0.43^***^	0.494^***^	0.37^***^
Overweight preoccupation	−0.10^**^	0.30^***^	−0.07	0.112^**^	0.01
Weight classification	0.08^*^	0.45^***^	−0.09^*^	−0.132^**^	−0.19^***^

* *p < 0.05*;

** *p < 0.01*;

*** *p < 0.001*.

A one-way between-subjects ANOVA was conducted to compare the effect of the Big Five personality traits on clothing style preferences. As seen in Table 3, there was a weak to moderate significant effect of extroversion [*F*_(4, 624)_ = 5.76, *p* = 0.0001], conscientiousness [*F*_(1, 624)_ = 6.80, *p* = 0.0001], agreeableness [*F*_(4, 624)_ = 4.86, *p* = 0.001], and openness to experience [*F*_(1, 624)_ = 7.38, *p* = 0.0001] on clothing styles. *Post-hoc* analyses using the Tukey *post-hoc* criterion for significance indicated that the average score of extroversion was significantly lower in the casual style condition (M = 3.45, SD = 0.74) than in the urban style condition (M = 3.8, SD = 0.63).

**Table 3:** One-way ANOVA for testing the Big Five personality traits and clothing styles (*N* = 792).

**Measure**	**Casual**	**Romantic**	**Dramatic**	**Urban**	**Classic**	***F***_**(4, 624)**_	**η** ^**2**^
	**M (SD)**	**M (SD)**	**M (SD)**	**M (SD)**	**M (SD)**		
Extraversion	3.45 (0.74)^a^	3.5 (0.75)	3.31 (0.89)	3.8 (0.64)^b^	3.54 (0.67)	5.76^***^	0.03
Conscientiousnes	3.83 (0.56)^a,b^	3.8 (0.64)^a,b^	3.6 (0.71)^a,d^	3.9 (0.54)^a,e^	4.04 (0.53)^a^	6.80^***^	0.04
Agreeableness	3.93 (0.49)^a,b^	3.92 (0.49)^a,c^	3.47(0.58)^a^	3.88 (0.75)^a,d^	3.89 (0.53)^a,e^	4.86^**^	0.03
Neuroticism	2.85 (0.79)	2.79 (0.81)	3.05 (0.71)	2.69 (0.75)	2.85 (0.76)	1.98	0.01
Openness	3.62 (0.59)^a,b^	3.78 (0.62)	3.57 (0.75)	3.9 (0.54)^a^	3.64 (0.50)^a,c^	7.38^***^	0.04

** *p < 0.01*;

*** *p < 0.001*.

a,b,c,d,e *Averages in a row with a different symbol differ significantly from each other in Tukey test*.

The average score of conscientiousness was higher in the classic style condition (M = 4.04, SD = 0.53) than in the dramatic style (M = 3.6, SD = 0.74) and the casual style (M = 3.83, SD = 0.56). The average score of agreeableness was lower in the dramatic style condition (M = 3.47, SD = 0.75) than all other styles of dress; casual style (M = 3.93, SD = 0.49), romantic style (M = 3.92, SD = 0.58), urban style (M = 3.88, SD = 0.53), and classic style (M = 3.89, SD = 0.59).

The average score of openness to experience was higher in the urban style condition (M = 3.9, SD = 0.54) than in the casual style condition (M = 3.62, SD = 0.59) and the classic style condition (M = 3.64, SD = 0.50).

### Body Image, Functions of Clothing, and Clothing Style

The pattern of correlations between body image dimensions and clothing functions is given in Table 2. As can be seen, appearance evaluation correlates negatively with using clothes for camouflage (*r* = −0.58, *p* < 0.001) and positively with other clothing functions: assurance (*r* = 0.34, *p* < 0.001), fashion (*r* = 0.28, *p* < 0.001), and individuality (*r* = 0.30, *p* < 0.001). Appearance orientation is positively correlated with assurance (*r* = 0.43, *p* < 0.001), fashion (*r* = 0.40, *p* < 0.001), and individuality (*r* = 0.37, *p* < 0.001). Weight preoccupation is positively correlated with camouflage (*r* = 0.30, *p* < 0.001). Weight classification is positively correlated with camouflage (*r* = 0.45, *p* < 0.001). The remaining correlations have a weak effect below *r* = 0.20 (see Table 2).

A one-way between-subjects ANOVA was conducted to compare the effect of body image dimensions on clothing style preferences. As seen in Table 4, there was a significant moderate effect of appearance orientation [*F*_(4,635)_ = 8.24, *p* = 0.0001] and appearance evaluation [*F*_(1,635)_ = 13.35, *p* = 0.0001] on clothing styles. *Post-hoc* analyses using the Tukey *post-hoc* criterion for significance indicated that the average score of appearance evaluation was lower in the casual style condition (M = 3.2, SD = 0.78) than in the urban style condition (M = 3.63, SD = 0.72). The average score of appearance orientation was higher in the urban style condition (M = 3.71, SD = 0.50) than in the dramatic (M = 3.25, SD = 0.71) and casual styles (M = 3.37, SD = 0.56).

**Table 4:** One-way ANOVA for testing body image dimensions and clothing styles (*N* = 792).

**Measure**	**Casual**	**Romantic**	**Dramatic**	**Urban**	**Classic**	***F***_**(4, 635)**_	**η** ^**2**^
	**M (SD)**	**M (SD)**	**M (SD)**	**M (SD)**	**M (SD)**		
App evaluation.	3.2 (0.78)^a^	3.52 (0.75)^a,b^	3.43 (0.70)	3.63 (0.72)^a,c^	3.45 (0.71)^a,d^	8.24^***^	0.04
App orient.	3.37 (0.56)^a^	3.61 (0.56)^a,b^	3.25 (0.72)^c,e^	3.71 (0.50)^a,c^	3.68 (0.58)^a,d,c,f^	13.36^***^	0.07
Weight pre.	2.81 (0.74)	2.9 (0.69)	3.0 (0.70)	2.89 (0.63)	2.9 (0.64)	1.13	0.0
Weight class.	3.4 (0.68)	3.33 (0.66)	3.4 (0.63)	3.25 (0.57)	3.42 (0.68)	2.19	0.0

*** *p < 0.001*.

a,b,c,d,e,f *Averages in a row with a different symbol differ significantly from each other in Tukey test*.

To further understand the relationship between clothing style preferences and body image, one more ANOVA was performed. A one-way between-subjects ANOVA to compare the effect of clothing functions on clothing style preference. As seen in Table 5, there was a significant moderate effect of comfort [*F*_(4, 662)_ = 12.8, *p* = 0.000], camouflage [*F*_(4, 662)_ = 8.1, *p* = 0.000], assurance [*F*_(4, 662)_ = 13.04, *p* = 0.000], fashion [*F*_(4, 662)_ = 39.0, *p* = 0.000], and individuality [*F*_(4, 662)_ = 44.67, *p* = 0.000] on the choice of clothing styles. *Post-hoc* analyses using the Tukey *post-hoc* criterion for significance indicated that the average score of all clothing functions was different in the casual style condition than in other clothing style conditions. Most relevant in the context of body image, the average score of camouflage was higher in the casual style (M = 3.32, SD = 0.71) than in the urban style condition (M = 2.89, SD = 0.71). The average score of assurance was lower in the casual style condition (M = 3.36, SD = 0.63) than in the urban style condition (M= 3.77, SD = 0.65).

**Table 5:** One-way ANOVA for testing clothing functions and clothing styles (*N* = 792).

**Measure**	**Casual**	**Romantic**	**Dramatic**	**Urban**	**Classic**	***F***_**(4, 662)**_	**η** ^**2**^
	**M (SD)**	**M (SD)**	**M (SD)**	**M (SD)**	**M (SD)**		
Comfort	4.27 (0.63)^a^	4.02 (0.72)	3.57 (1.03)^a,b^	3.86 (0.75)^a,c^	3.97 (0.75)^a,d^	12.8^***^	0.07
Camouflage	3.32 (0.71)^a^	3.03 (0.77)	3.17 (0.93)	2.89 (0.71)^a,b^	3.13 (0.80)	8.10^***^	0.04
Assurance	3.36 (0.63)^a^	3.63 (0.60)	3.20 (0.79)^a,b^	3.77 (0.65)^a,c^	3.56 (0.62)^a,d^	13.04^***^	0.07
Fashion	2.5 (0.78)^a^	3.2 (0.84)^a,b^	3.41 (0.65)^a,c^	3.43 (0.77)^a,d^	3.18 (0.81)^a,e^	39.0^***^	0.1
Individuality	2.65 (0.76)^a^	3.24 (0.75)^a,b^	3.44 (0.89)^a,c^	3.65 (0.68)^a,d^^;^^d,e^	3.09 (0.77)^a,e^	44.67^***^	0.2

*** *p < 0.001*.

a,b,c,d,e *Averages in a row with a different symbol differ significantly from each other in Tukey test*.

Finally, backwards stepwise logistic regressions were preformed to explore the effect of the different variables on each style of dress. Significant results were found in the regression model for predicting choices of urban style vs. casual style.

As seen in Table 6, variables that predict urban style of dress, vs. casual style, are openness (beta = 0.58; *p* = 0.03), fashion (beta = 0.72; *p* = 0.00) individuality (beta = 1.38; *p* = 0.00), lower levels of comfort (beta = −0.71; *p* = 0.00), and lower camouflage (beta = −0.67, *p* = 0.00). Thus, women who are more open to experience (OR = 1.8; IC 95%: 1.05–3.0), who seek fashion (OR = 2.05; IC 95%: 1.37–3.05) and individuality (OR = 3.96; IC 95%: 2.46–6.3) are more likely to exhibit an urban style of dress. These women are less motivated by comfort (OR = 0.49; IC 95%: 0.31–0.77) and camouflage (OR = 2.05; IC 95%: 1.37–3.05) when they chose their clothing style.

**Table 6:** Multiple logistic regression analysis to predict urban style vs. casual style of dress (*N* = 378).

**Predictor**	**B**	**OR**	**CI (OR)**
Openness to experience	0.58^*^	1.8	1.05–3.0
Comfort	−0.71^**^	0.49	0.31–0.77
Camouflage	−0.67^**^	0.5	0.33–0.78
Fashion	0.72^***^	2.05	1.37–3.05
Individuality	1.38^***^	3.96	2.46–6.3

* *p < 0.05*;

** *p < 0.01*;

*** *p < 0.001*.

*Likelihood Ratio test: χ^2^(5) = 193.32, p < 0.0001*.

## Discussion

This study explored the relationships between clothing practices to personality traits and body image among Israeli women. Overall, the results supported the hypothesis that clothing practices are related to personality traits and can be predicted by body image.

Using the Big Five personality traits model, this study found that conscientiousness was related with a classic style of dress, defined as formal, conventional, and representative clothing. The Big Five model describes people with high levels of conscientiousness as organized, reliable, punctual and neat (Costa and McCrae, 1992), and wearing formal clothing was found to support a self-perception of neatness, cultivation, and restraint (Hannover and Kühnen, 2002). This research also found conscientiousness to be negatively correlated with camouflage and positively correlated with assurance, fashion, and individuality. These findings correspond with previous findings that people who wear formal clothes perceive themselves as highly competent, trustworthy, and authoritative (Peluchette and Karl, 2007).

This study found that agreeableness was lowest among women who identified with the dramatic style, defined as unusual and unique style of dress. The Big Five model describes people with high levels of agreeableness as warm, kind a cooperative. They are more motivated by solidarity than assertiveness or excitement seeking (Costa and McCrae, 1992, 2009).

Extroversion was related to an urban style of dress, defined as an eclectic and playful style characterized by creative combinations of clothes. These findings correspond with the Big Five model perception of extroverts as sociable, people-oriented, active, optimistic, and fun loving (Costa and McCrae, 1992). Moreover, extroverts were found to prefer exciting fashion brands that are typically perceived as active, adventurous, and cool (Mulyanegara et al., 2009), characterizations that resonate with the definition of urban style.

The present research indicates that camouflage is a function characteristic of the casual clothing style, a minimalist style featuring jeans and t-shirts. It was correlated with high levels of camouflage, low levels of extroversion, and low openness to experience, suggesting that women who identified with this style were the most introverted and conventional thinking among the research sample.

Openness to experience was highest among women who identified with the urban style. The Big Five model describes people with high levels of openness to experience as being curious, creative, and untraditional, and as having broad interests (Costa and McCrae, 1992). Women who identified with the urban style were more open-minded and creative than those wearing the casual style. Moreover, body image played an important role in the choice between the urban and the casual style.

The final logistic regressions reinforced this distinction; When comparing the prediction to choose urban vs. casual style, women who choose urban are less preoccupied by the need to camouflage their bodies or what they define as comfortable clothing. They are interested in fashion and individuality, i.e., using clothes as a tool for self-expression, rather than an adjustment to cultural beauty ideals.

Women who identified with the urban style, a creative and expressive style of dress, were more likely to feel confident with their bodies. They also tended to be extroverted, which echoes research that shows extroversion to be correlated with a higher appreciation of one's own body (Swami et al., 2012). In contrast, women who identified with the casual style were distinguished by higher levels of camouflage and lower assurance. This corresponds with Trautmann et al. (2007) findings that women who were more dissatisfied with their bodies were more likely to camouflage their bodies with dark-colored and baggy tops, and avoid wearing revealing, brightly colored, or tightly fitting clothing.

The present research reinforces previous findings regarding the relationship between body image and clothing practices. Appearance evaluation was negatively correlated with camouflage and appearance orientation was positively correlated with fashion. That is, the better women feel about their bodies, the higher is their ability to use clothes for self-expression and enjoyment. These findings align with Tiggemann and Andrew's (2012) research on the interrelationships between women's attitudes toward clothing and their attitude toward their bodies.

The present study showed that weight preoccupation and weight classification correlated with camouflage. These results are in line with Kwon and Parham (1994) findings that women select clothes more for camouflage and less for individuality when feeling “fat.” Since there were no BMI differences among the different groups, and BMI was not a predictor to any style of dress, the urban style seems to foster body-positive clothing practices. As indicated by the logistic regression, openness to experience may play an important role. Openness to experience was estimated to increase the odds to choose an urban clothing style (vs. casual style) by 80%.

Openness to experience is associated with non-conformity (Feist and Brady, 2004), suggesting that these women are able to enjoy and play with their clothes despite Western society's pressures to conform to a strict beauty standard and conceal possible “imperfections.”

Openness to experience is also related to psychological flexibility, including body image flexibility. Having a flexible body image decreases body dissatisfaction and increases flexible responses to body-related thoughts and feelings (Sandoz et al., 2013). A lack of flexibility may then drive women's tendency to choose concealing clothes when they see themselves as “fat” (Trautmann et al., 2007).

It is likely that women who identified with the urban style are more flexible both in their body image and their clothing practices. The urban style group may represent what Cash (2008) defines as “flexible groomers,” individuals characterized by a playful and enjoyable use of clothing styles, fabrics, colors, cosmetics, hairstyles, jewelry, and fragrances. Flexible groomers use grooming for mastery and pleasure, and not in a rigid effort to maintain positive appearance.

## Conclusions

This study highlights the relationship between clothing practices and body image among women. It indicates that personality traits play a role in clothing choices, which suggests that one's clothing is a kind of manifestation of the self (e.g., Sontag and Lee, 2004).

Openness to experience may foster body-positive clothing practices that are oriented toward self-expression and individuality rather than camouflage. In this sense, their choice of clothing can help women overcome objectification and cultural body-ideal pressures (Fredrickson and Roberts, 1997) by enabling them to dress for their own validation and pleasure. This can be used in clinical practice by encouraging women to experience and play with their clothes—thereby facilitating a more flexible body image and lessening their rigid perception of clothing practices.

The ever-increasing cultural pressure to attain the ideal body highlights the importance of understanding the role of clothing practices in fostering positive body image. Moreover, the relationship between clothing practices and personality traits sheds light on the psychology of dress, a generally neglected field of research.

## Limitations

The shortcomings of this research are the following: First, the definition of clothing styles was designed for this research, and subjects were asked to identify only one style of dress that is most relevant to them. This self-report measure was the most reliable measure found for this research, based on the existing research in this field of knowledge, but its reliability is limited. Second, the research is based on a convenience sample, only female subjects were included, and they were predominantly non-religious, financially secure, and well-educated. Further research is needed to understand clothing practices among broader populations.

## Data Availability Statement

The original contributions presented in the study are included in the article/supplementary material, further inquiries can be directed to the corresponding author.

## Ethics Statement

Ethical approval was not provided for this study on human participants because all subjects voluntarily and willingly filled self report online questionnaires. Written informed consent for participation was not required for this study in accordance with the national legislation and the institutional requirements.

## Author Contributions

The author confirms being the sole contributor of this work and has approved it for publication.

## Conflict of Interest

The author declares that the research was conducted in the absence of any commercial or financial relationships that could be construed as a potential conflict of interest.

## Publisher's Note

All claims expressed in this article are solely those of the authors and do not necessarily represent those of their affiliated organizations, or those of the publisher, the editors and the reviewers. Any product that may be evaluated in this article, or claim that may be made by its manufacturer, is not guaranteed or endorsed by the publisher.
