# Corrigendum: Styling the Self: Clothing Practices, Personality Traits, and Body Image Among Israeli Women

**DOI:** 10.3389/fpsyg.2021.789720

**Published:** 2021-11-02

**Authors:** Tali Stolovy

**Affiliations:** ^1^Academic College of Society and the Arts, Netanya, Israel; ^2^Emili Sagol Creative Arts Therapies Research Center, Faculty of Social Welfare and Health Sciences, University of Haifa, Haifa, Israel

**Keywords:** personality traits, body image, women–clothing, clothing styles, clothing practices

In the original article, there was an error. There were two language mistakes in the abstract. The redundant word “are” in line 6 was deleted. The word “a” in line 8 was corrected to “an”. A correction has been made to *Abstract*. The corrected paragraph is shown below.

Research has shown that women tend to use clothes to present or disguise their bodies and that clothing practices can be predicted by body image. This study explored the relationships between clothing practices, personality traits, and body image among Israeli women, using the Big Five personality traits model (NEO-FFI) and a body image measure (MBSRQ) to explore clothing styles and practices among Israeli women (*N* = 792, Mean age = 42.19). It found that women with more openness to experience (OR = 1.8; IC 95%: 1.05–3.0), who seek fashion (OR = 2.05; IC 95%: 1.37–3.05) and individuality (OR = 3.96; IC 95%: 2.46–6.3) are more likely to exhibit an urban, sophisticated style of dress. These women are less motivated by comfort (OR = 0.49; IC 95%: 0.31–0.77) and camouflage (OR = 2.05; IC 95%: 1.37–3.05), that are associated with casual, minimalist style of dress. This study indicates that openness to experience may foster body-positive clothing practices. In this way, their choice of clothing can help women overcome objectification and cultural body-ideal pressures, promoting self-validation and mastery.

The authors apologize for this error and state that this does not change the scientific conclusions of the article in any way. The original article has been updated.

## Publisher's Note

All claims expressed in this article are solely those of the authors and do not necessarily represent those of their affiliated organizations, or those of the publisher, the editors and the reviewers. Any product that may be evaluated in this article, or claim that may be made by its manufacturer, is not guaranteed or endorsed by the publisher.

